# Cost-effectiveness of screening of coronary artery disease in patients with type 2 diabetes at a very high cardiovascular risk (SCADIAB) study. Updated study design: emulation of a randomised target trial using electronic health records

**DOI:** 10.1186/s12933-023-02069-y

**Published:** 2023-11-28

**Authors:** Kamel Mohammedi, Audrey Cordon, Vincent Rigalleau, Ninon Foussard, Céline Bairras-Martin, Thierry Couffinhal, Julien Bezin, Antoine Benard

**Affiliations:** 1 Univ. Bordeaux, INSERM, BMC, U1034, 33600 Pessac, France; 2 Department of Endocrinology, Diabetes and Nutrition, Bordeaux University Hospital, Hôpital Haut-Lévêque, Avenue de Magellan, 33604 Pessac Cedex, France; 3 Clinical Epidemiology Unit (USMR), CIC-EC 14-01, Bordeaux University Hospital, Bordeaux, France; 4 INSERM U1219-Bordeaux Population Health Research Center, Bordeaux, France; 5 Internal Promotion Department, Bordeaux University Hospital, DCRI, Talence, France; 6 Department of Cardiology, Bordeaux University Hospital, Hôpital Haut-Lévêque, Pessac, France; 7 INSERM, Bordeaux Population Health Research Center, U1219, Team Pharmacoepidemiology, Bordeaux, France; 8 Department of Pharmacology, Bordeaux University Hospital, Bordeaux, France; 9 INSERM, Bordeaux Population Health Research Center, U1219, Team EMOS, Bordeaux, France

## Background

Type 2 diabetes mellitus (T2DM) is a substantial health problem with a high clinical and economic burden [1, 2]. Despite a recent prognostic improvement, coronary artery disease (CAD) remains one of the leading causes of mortality in people with T2DM [3]. CAD is often severe and silent in patients with T2DM [4], prompting screening in asymptomatic individuals. Previous randomised controlled trials (RCTs) have failed to provide evidence of the effectiveness of routine CAD screening in reducing major cardiovascular events and mortality in people with T2DM at moderate to high cardiovascular risk [5–9]. However, doubts remain about T2DM patients at a very high cardiovascular risk, who can benefit from this screening strategy, but in whom trials have not yet been conducted. Despite this lack of concrete evidence, current clinical practice and guidelines recommend routine CAD screening for silent CAD in individuals with diabetes at high cardiovascular risk [10]. The widespread practice CAD screening results in significant healthcare costs due to invasive testing, revascularisation procedures, and intensified pharmacological therapies. The cost-effectiveness of routine CAD screening has not been prospectively evaluated in T2DM people. Only two economic studies based on Markov models have addressed this issue by comparing screening versus no screening strategy in Japanese and American populations [11, 12]. The results of these studies were inherently limited by assumptions inherent in the models, uncertainties in the epidemiological and cost data, and variations in health care costs between countries.

In the midst of this discrepancy between the lack of tangible evidence and clinical practice, we are conducting the Cost-effectiveness of Screening of Coronary Artery disease in patients with type 2 DIABetes at a very high cardiovascular risk (SCADIAB) study to estimate the cost-effectiveness of routine CAD screening in T2DM patients with a very high cardiovascular risk.

### Study design

The study design and protocol have been published in a previous issue of this journal [13]. We report here updated methods and statistical approach that will be performed in this study, namely the emulation of a 2-arm randomised target trial using the French electronic health record (SNDS, *Système National des Données de Santé*), a claim database encompassing the whole French population [14, 15].

### Recruitment of participants and follow‑up

**Fig. 1:**
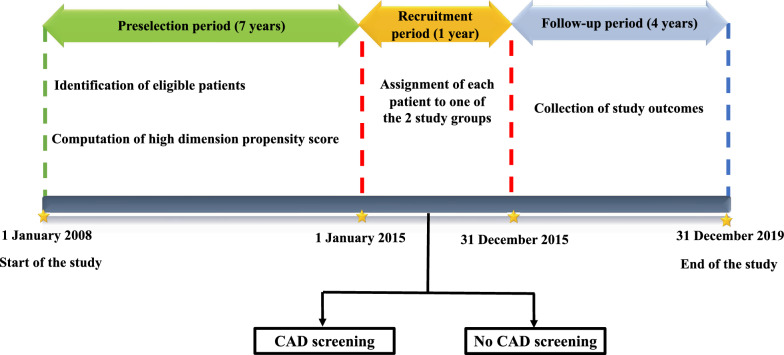
Study design

Data will be extracted from 1 January 2008 to 31 December 2019. The screening period will cover the 7 years from 1 January 2008 to 31 December 2014, which will allow us to identify patient eligibility criteria. All patients identified in the SNDS databases during the pre-selection period who met the inclusion criteria will be recruited in the study from 1 January 2015 to 31 December 2015. Participants will be followed for 4 years, until 31 December 2019 (Fig. 1).

### Screening strategies and study allocation

Each patient will be assigned into one of the two study groups according to the CAD screening strategy performed during the recruitment period. Patients who had at least one CAD screening test will be assigned to the routine CAD screening group and included at the time of the first CAD test. Patients who did not perform any CAD screening (except for the resting electrocardiogram) will be assigned to the control group, and included at the time of the resting electrocardiogram, or at the time of a medical visit (general practitioner, diabetologist, or cardiologist), whichever comes first.

### Eligibility criteria

The key inclusion criteria are patients aged 40 years or older, with T2DM for at least 7 years, two or more additional cardiovascular risk factors (obesity, hypertension, hypercholesterolemia, or tobacco smoking), and at least one microvascular or macrovascular disease: carotid stenosis, transient ischemic attack (TIA), stroke, lower-limb peripheral artery disease, chronic kidney disease (CKD), severe diabetic retinopathy requiring laser photocoagulation, or peripheral or autonomic diabetic neuropathy. The main exclusion criteria include the presence of CAD (a history of acute coronary syndrome, coronary revascularization, angina pectoris, or unstable angina), the performance of one or more CAD screening tests, or any visit to an emergency department for chest pain resulting for admission to a cardiac intensive care unit.

### Outcomes

The primary outcome is the incremental cost per life-year gained over 4 years in CAD routine screening group versus no screening in the perspective of French health insurance. The key secondary health economic endpoints include the cost-consequence analysis linking the total 4-year direct costs of each strategy and clinical endpoints; an analysis of budget impact for the French Insurance system to promote the routine CAD screening; and the total care consumption during follow-up. The key secondary clinical endpoints included the effects of CAD screening (versus no screening) in terms of major adverse cardiovascular events (a composite of acute coronary syndrome, coronary revascularization, or hospitalization for heart failure); all-cause mortality; major cerebrovascular events (a composite of stroke, hospitalization for TIA, or carotid revascularization); major adverse limb events (a composite of ischemic diabetic foot, lower-limb revascularization or amputation); and CKD or end-stage kidney disease (ESKD, requirement of any sustained haemodialysis or kidney transplantation). Each component of these composite endpoints will also be considered individually. Major cerebrovascular events, major adverse limb events and ESKD will be assessed during follow-up among participants without a baseline history of each condition (as appropriate). Finally, SCADIAB will also assess the frequency of routine CAD screening, expressed as the number of examinations performed yearly per individual.

### Causal effects of interest

The primary causal effect in our study is the intention-to-treat effect. That is the comparative effect of being assigned to the CAD screening strategies at inclusion, regardless of whether the individuals continue following the strategies during follow-up. The per-protocol effect will also be investigated after exclusion of participants from the control group who underwent routine CAD screening during follow-up.

### Statistical analysis

The gross estimate of the incremental cost per life-year gained at 4 years will be conducted. High dimension propensity score will be used to balance the baseline characteristics of participants. Comparisons between study groups will be performed without adjustment; and with adjustment for confounding factors using the weighted by inverse probability of receiving treatment (IPTW). A cost-consequence analysis will be performed linking results of all direct costs, and results on major cardiovascular events. A budget impact analysis will be conducted to determine the public expenses of the spread of a routine CAD screening at 5 years from French health insurance perspective. The risk of clinical outcomes by study groups will be assessed using IPTW proportional hazards survival regression models. We will also consider the competing risk of all-cause death.

We will perform subgroup analyses by sex, age and frequency of CAD tests. Finally, a sensitivity analysis is planned to assess the interest of routine CAD screening in T2DM people with at least 2 cardiovascular risk factors without microvascular or macrovascular disease.

### Study progress

The study protocol has been approved by the French ethics authorities (as detailed in the Declaration section). In November 2023, the steering committee made an amendment to use a target trial approach, which was approved by the ethics authorities. Analysis will start before the end of 2023, and the main results will be published in 2024.

## Conclusion

SCADIAB will use a target trial design to assess the cost-effectiveness of the routine CAD screening strategy in T2DM patients at a very high-risk for cardiovascular disease. SCADIAB will offer valuable insights for healthcare resource allocation and clinical decision-making.

## Data Availability

Not applicable.

## Abbreviations

CAD: Coronary artery disease
CKD: Chronic kidney disease
ESKD: End-stage kidney disease
IPTW: Inverse probability of receiving treatment weighting
RCT: Randomized controlled trial
SCADIAB: Cost-effectiveness of Screening of Coronary Artery disease in patients with type 2 DIABetes at a very high cardiovascular risk
SNDS: Système National des Données de Santé (The French National Health Data System)
T2DM: Type 2 diabetes mellitus
TIA: Transient ischemic attack
